# Editorial: Advanced Functional Materials Derived From One-Dimensional Clay Minerals

**DOI:** 10.3389/fchem.2021.821964

**Published:** 2021-12-10

**Authors:** Aiqin Wang, Bin Mu, Giuseppe Lazzara, Yunfei Xi

**Affiliations:** ^1^ Lanzhou Institute of Chemical Physics (CAS), Lanzhou, China; ^2^ Department of Physics and Chemistry, University of Palermo, Palermo, Italy; ^3^ School of Earth, Environmental and Biological Sciences, Queensland University of Technology, Brisbane, QLD, Australia

**Keywords:** palygorskite, attapulgite, sepiolite, functional materials design, halloysite

**FIGURE 1:**
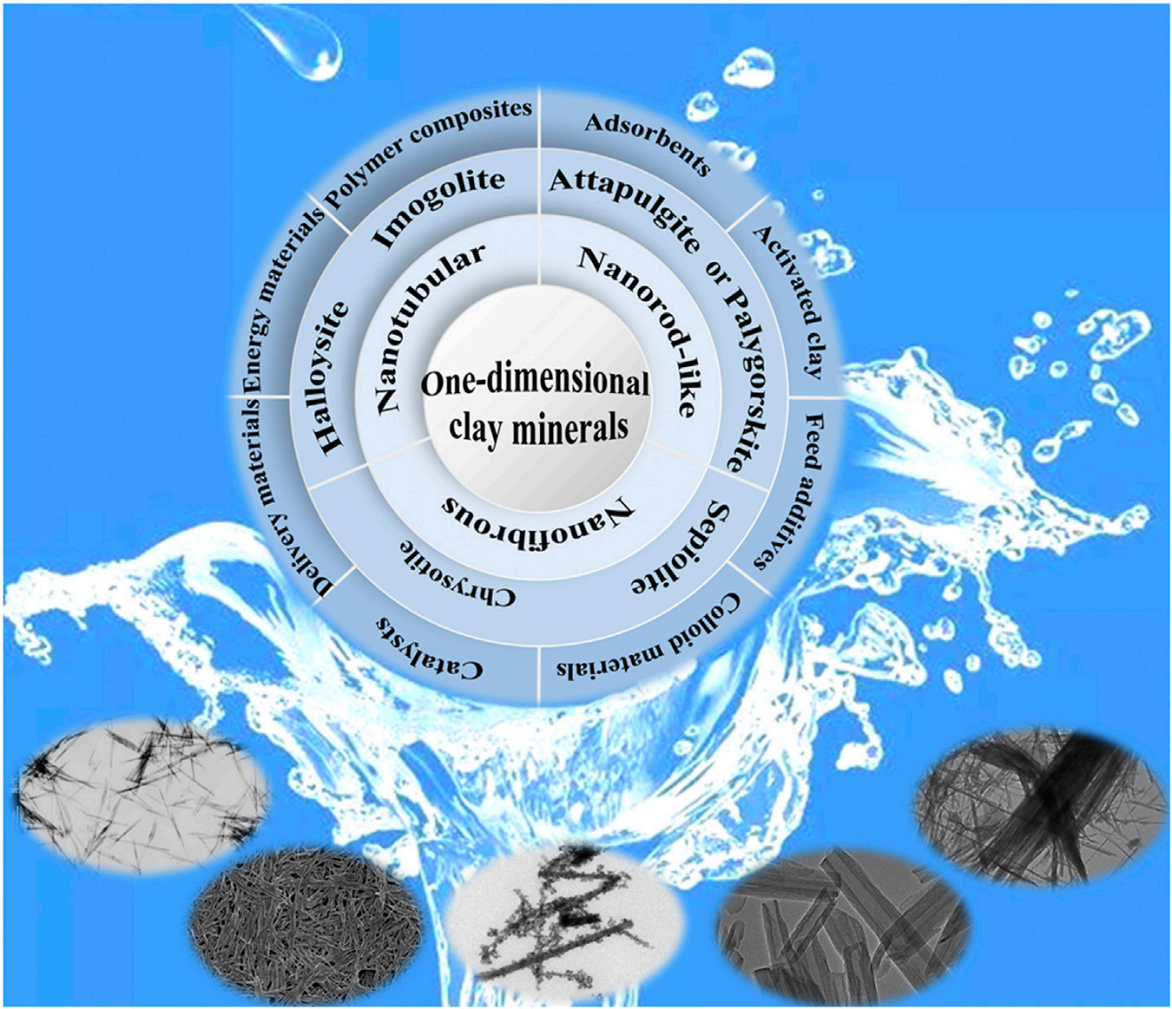
Advanced functional derived from one-dimensional clay minerals.

Due to the abundance in nature, unique structural characteristics, low-cost and environmental friendliness, different target objects can be incorporated into one-dimensional clay minerals by surface modification or structural transformation to prepare functional materials (Figure 1). Thus one-dimensional clay minerals have been one of the research focuses of material and environmental sciences, chemistry and chemical engineering, biomedicine and mineralogy. This Research Topic collects the latest research progress on the green functional composites based on one-dimensional clay minerals, especially sepiolite and palygorskite with one-dimensional fibrous or rod-like morphologies and zeolite-like channels. Sepiolite nanofibers could be served as natural green carriers to load zero-valent iron or ZnFe_2_O_4_ nanoparticles for catalytic degradation of hazard antibiotics in aqueous solution, and the removal ratio of tetracycline hydrochloride was above 92%, and thus the as-prepared composites presented great potential for treatment of antibiotic wastewater. In addition, sepiolite-hydrogels were fabricated by a facile ultrasound irradiation-assisted dispersion method, and then the composite hydrogels were employed as rheological additives to design functional clay-based nanoarchitectured materials combining with kaolinite and halloysite aluminosilicates, as well as synthetic Mg, Al-layered double hydroxide. In order to enhance the loading content and bonding ability of sepiolite toward indigo molecules, sepiolite was hydrothermally modified with Al^3+^ to realize the partial lattice ions substitution, which provided a promising strategy for regulating the structure and properties of clay minerals to prepare composite pigment and functional materials. Compared with sepiolite, palygorskite has the small nanochannels (0.37 nm × 0.64 nm), and it is more suitable for fabrication of Maya blue or Maya blue-like pigments with excellent stability and weather-resistance. In order to adjust and enrich the colors of palygorskite-based hybrid pigments, different metal ions were introduced to prepare curcumin/palygorskite hybrid pigments with different colors and good antioxidant activity. The color change and stability enhancement were attributed to the coordination of metal ions with curcumin and Si-OH of palygorskite, and the hydrogen bond interaction with complexion water confined in the nanochannels of palygorskite. In conclusion, different functional materials were designed and fabricated *via* various physical and chemical technologies based on the one-dimensional nanostructure and unique nanochannels of one-dimensional clay minerals.

With the increase in the understanding of structure and physicochemical properties of one-dimensional clay minerals, more high-performance functional materials will be developed to be adsorbents, colloidal materials, hybrid materials, polymeric composites, bio-inspired materials, catalysts and energy materials, etc. according to their adsorption, colloid, carrier and reinforcing functions. However, it is worth noting that green preparation methods and the roles of the associated clay minerals should be taken into account for preparation of functional materials using one-dimensional clay minerals, or even other clay minerals. This Research Topic has collected some recent advances on the relevant research, and it is convenient for the readers to know the latest research progress on the one-dimensional clay minerals-based functional materials. What’s more, it also attracts much attention to design the green and eco-friendly functional materials derived from natural clay minerals by green non-pollution technology.

